# Hemiptera of Canada

**DOI:** 10.3897/zookeys.819.26574

**Published:** 2019-01-24

**Authors:** Robert G. Foottit, H. Eric L. Maw, Joel H. Kits, Geoffrey G. E. Scudder

**Affiliations:** 1 Agriculture and Agri-Food Canada, Ottawa Research and Development Centre and Canadian National Collection of Insects, Arachnids and Nematodes, K. W. Neatby Bldg., 960 Carling Ave., Ottawa, Ontario, K1A 0C6, Canada Agriculture and Agri-Food Canada Ottawa Canada; 2 Department of Zoology and Biodiversity Research Centre, University of British Columbia, 6270 University Boulevard, Vancouver, British Columbia, V6T 1Z4, Canada University of British Columbia Vancouver Canada

**Keywords:** Barcode Index Number (BIN), biodiversity assessment, Biota of Canada, DNA barcodes, Hemiptera, true bugs

## Abstract

The Canadian Hemiptera (Sternorrhyncha, Auchenorrhyncha, and Heteroptera) fauna is reviewed, which currently comprises 4011 species, including 405 non-native species. DNA barcodes available for Canadian specimens are represented by 3275 BINs. The analysis was based on the most recent checklist of Hemiptera in Canada (Maw et al. 2000) and subsequent collection records, literature records and compilation of DNA barcode data. It is estimated that almost 600 additional species remain to be discovered among Canadian Hemiptera.

The order Hemiptera, the true bugs, is a relatively large order. Worldwide there are an estimated 106,970 described species (Henry 2017, Bartlett et al. 2018, Hardy 2018). The recognised Canadian Hemiptera fauna (Table 1) has been greatly expanded since the review by Scudder (1979), with an increase of 937 species above the 3079 then known. The checklist of Canadian Hemiptera (Maw et al. 2000) provided comprehensive lists and distributions for all species recognised at that time. Here we present updated totals, including a number of additional unpublished records represented by specimens in the Canadian National Collections of Insects, Arthropods and Nematodes (CNCI) in Ottawa. Gwiazdowski et al. (2015) presented a detailed analysis of all Hemipteran DNA barcodes available for the Canadian fauna.

**Table 1.** Census of Hemiptera in Canada.

Taxon^1^	No. species reported in Scudder (1979)^2^	No. species currently known from Canada^3^	No. BINs^4^ available for Canadian species	Est. no undescribed or unrecorded species in Canada	General distribution by ecozone^4A^	Information sources^5^
**Suborder Sternorrhyncha**						
**Superfamily Psylloidea**						CNCI
Aphalaridae **^6^**	50	37 (1)	19	10	all ecozones but Arctic	
Calophyidae **^6^**	?	1	0	0	Mixedwood Plains	
Liviidae **^6^**	4	14 (3)	6	2	all ecozones but Arctic	
Psyllidae **^6^**	35	52 (5)	65	20	all ecozones but Arctic	
Triozidae	18	25 (1)	13	10	all ecozones but Arctic	
**Superfamily Aleyrodoidea**						
Aleyrodidae	3	13 (4)	40	40	all ecozones south of taiga	CNCI
**Infraorder Aphidomorpha**						
**Superfamily Adelgoidea**						
Adelgidae	22	18 (5)	14	1	all ecozones but Arctic	
**Superfamily Aphidoidea**						
Aphididae	650	847 (164)	758	100	all ecozones	CNCI
**Superfamily Phylloxeroidea**						
Phylloxeridae	6	8 (2)	11	4	Pacific Maritime, Mixedwood Plains, Boreal Plains (1 sp.)	
**Infraorder Coccomorpha**						
**Superfamily Coccoidea**						
Asterolecaniidae	1	2 (2)	0	0	Pacific Maritime, Mixedwood Plains	
Coccidae	15	26 (12)	7	5	all ecozones, mostly south of Arctic	
Cryptococcidae	?	2 (2)	0	0	Mixedwood Plains, Atlantic Maritime	
Dactylopiidae	1	1	0	0	Prairies	
Diaspididae	16	30 (10)	4	10	all ecozones, mostly south of taiga	
Eriococcidae	3	3 (2)	2	0	Pacific Maritime, Mixedwood Plains, Atlantic Maritime	
Kermesidae	0	4	0	1	Mixedwood Plains	
Margarodidae	4 **^7^**	0	0	0		
Matsucoccidae **^7^**	?	2	0	0	Mixedwood Plains, Atlantic Maritime	
Ortheziidae **^8^**	3	5	2	0	all ecozones but Arctic	
Pseudococcidae	13 **^9^**	25 (9)	11	10	all ecozones, mostly south of taiga	Newton et al. 2011; CNCI
Putoidae **^9^**	?	2	0	0	Montane Cordillera, Boreal Cordillera	
Rhizoecidae	0	1	0	2	Pacific Maritime	CNCI
Steingeliidae **^7^**	?	1 (1)	0	0	Mixedwood Plains	
Xylococcidae **^7^**	?	1	0	0	Atlantic Maritime	
**Total Sternorrhyncha**	**844**	**1120 (223)**	**955**	**215**		
**Suborder Auchenorrhyncha**						
**Superfamily Fulgoroidea**						
Acanaloniidae **^10^**	?	2	2	0	Mixedwoods Plains	
Achilidae	17	19	15	3	all ecozones but Arctic	
Caliscelidae	7	11	7	0	mostly south of boreal	
Cixiidae	25 **^11^**	31	14	5	all ecozones but Arctic	
Delphacidae	81	138 (1)	102	30	all ecozones	
Derbidae	14	21	17	3	all ecozones south of taiga; most in Mixedwood Plains	
Dictyopharidae **^12^**	4	8	5	1	widespread south of taiga	
Flatidae	1	3	3	0	Mixedwoods Plains	
Issidae	3 **^10^**	2	1	0	Mixedwoods Plains	
Kinnaridae **^11^**	?	1	0	0	Western Interior Basin	
**Infraorder Cicadomorpha**						
**Superfamily Cicadoidea**						
Cicadidae	9	21	10	7	mostly south of taiga, Taiga Plains (1 sp.)	
**Superfamily Cercopoidea**						
Aphrophoridae **^13^**	?	23 (4)	19	2	all ecozones but Arctic	
Cercopidae	33 **^13^**	1	0	0	Mixedwoods Plains	
Clastopteridae **^13^**	?	12	8	2	all ecozones south of taiga	
**Superfamily Membracoidea**						
Cicadellidae	800	1097 (76)	1144	150	all ecozones	Dmitriev 2018, specimens in CNCI
Membracidae	70	101 (1)	65	20	widespread south of boreal, few in Boreal Shield and Boreal Plains	Dmitriev 2018
**Total Auchenorrhyncha**	**1064**	**1491 (82)**	**1412**	**223**		
**Suborder [?] Heteroptera**						Scudder 2008 and references included therein, Scudder 2012, Roch 2017
**Infraorder Enicocephalomorpha**						
**Superfamily Enicocephaloidea**						
Aenictopecheidae	0	1	0	0	Boreal Plains (single collection)	
Enicocephalidae **^14^**	1	1	2	1	Mixedwood Plains, Atlantic Maritime	
**Infraorder Dipsocoromorpha**						
**Superfamily Dipsocoroidea**						
Ceratocombidae **^15^**	1	1	7	5	Pacific Maritime, Mixedwood Plains	
Schizopteridae	0	1 (1)	0	0	Pacific Maritime	
**Infraorder Gerromorpha**						
**Superfamily Gerroidea**						
Gerridae	19	22	13	1	all ecozones but Arctic	
Veliidae	6	8	6	0	all ecozones south of taiga	
**Superfamily Hebroidea**						
Hebridae	4	5	2	0	all ecozones south of taiga	
**Superfamily Hydrometroidea**						
Hydrometridae	1	1	1	0	Pacific Maritime, Prairies, Mixedwood Plains, Atlantic Maritime	
**Superfamily Mesovelioidea**						
Mesoveliidae	2	2	2	0	all ecozones south of taiga	
**Infraorder Nepomorpha**						
**Superfamily Corixoidea**						
Corixidae	72	79	57	2	all ecozones but Arctic	
**Superfamily Naucoroidea**						
Naucoridae	0	1	1	0	Mixedwood Plains	
**Superfamily Nepoidea**						
Belostomatidae	3	4	2	0	all ecozones but Arctic	
Nepidae	7	4	6	1	Mixedwood Plains, southern Boreal Shield	
**Superfamily Notonectoidea**						
Notonectidae	12	12	10	0	all ecozones but Arctic	
Pleidae	2	1	1	0	Mixedwood Plains, southern Boreal Shield	
**Superfamily Ochteroidea**						
Gelastocoridae	1	1	1	0	Pacific Maritime, Prairies, Mixedwood Plains	
Ochteridae	0	1	0	0	Mixedwood Plains	
**Infraorder Leptopodomorpha**						
**Superfamily Saldoidea**						
Saldidae	36	38	23	2	All ecozones, mostly south of Arctic	
**Infraorder Cimicomorpha**						
Cimicoidea						
Anthocoridae	41^16^	39 (7)	36	1	widespread south of taiga	
Cimicidae	4	7	3	0	widespread south of taiga	
Lasiochilidae **^16^**	?	1	0	0	Mixedwood Plains	
Lyctocoridae **^16^**	?	6	2	0	all ecozones south of taiga	
**Superfamily Naboidea**						
Nabidae	12	22 (3)	19	2	all ecozones south of Arctic	
**Superfamily Microphysoidea**						
Microphysidae	0	3 (3)	1	0	near Pacific and Atlantic ports of entry	
**Superfamily Miroidea**						
Miridae	601**^17^**	706 (57)	414	100	mostly south of Arctic, widespread	
Tingidae	46	52 (6)	30	10	all ecozones south of taiga	
**Superfamily Reduvioidea**						
Reduviidae	26**^18^**	29 (3)	20	0	all ecozones south of taiga	
**Infraorder Pentatomomorpha**						
**Superfamily Aradoidea**						
Aradidae	47 **^19^**	51	13	2	all ecozones but Arctic	
**Superfamily Coreoidea**						
Alydidae	10	9	7	0	all ecozones but Arctic	
Coreidae	11	15	14	0	all ecozones but Arctic	
Rhopalidae	9	19 (1)	14	2	all ecozones but Arctic	
**Superfamily Lygaeoidea**						
Artheneidae	0	1 (1)	1	0	all ecozones south of taiga	
Berytidae **^20^**	3	5 (1)	6	0	all ecozones south of boreal	
Blissidae **^21^**	?	6	4	0	all ecozones south of boreal	
Cymidae **^21^**	?	5	4	0	all ecozones south of taiga	
Geocoridae **^21^**	?	10	5	0	all ecozones but Arctic	
Heterogastridae **^21^**	?	2 (1)	0	0	Pacific Maritime	
Lygaeidae	100 **^21^**	27 (1)	18	2	all ecozones, most south of Arctic	
Oxycarenidae **^21^**	?	5 (1)	1	0	all ecozones but Arctic	
Pachygronthidae **^21^**	?	3	4	1	all ecozones but Arctic	
Piesmatidae	1	4	1	0	all ecozones south of taiga	
Rhyparochromidae **^21^**	?	71 (10)	52	7	all ecozones but Arctic	
**Superfamily Pentatomoidea**						
Acanthosomatidae	5	5 (1)	3	0	all ecozones but Arctic	
Cydnidae	7	12 (1)	13	3	Pacific Maritime, Mixedwood Plains	
Pentatomidae	63 **^22^**	77 (1)	68	5	all ecozones but Arctic	
Scutelleridae	9	13	9	2	all ecozones but Arctic, mostly south of taiga	
Thyreocoridae **^23^**	9	11	12	2	all ecozones but Arctic, mostly south of taiga	
**Total Heteroptera**	**1171**	**1400 (100)**	**908**	**151**		
**Total Hemiptera**	**3079**	**4011 (405)**	**3275**	**589**		

**^1^**Classification follows Ouvrard (2018, Psylloidea), Favret (2018, Aphidomorpha), García Morales et al. (2016, Coccoidea), Bartlett et al. (2018, Auchenorrhyncha), and Henry (2017, Heteroptera). **^2^**Some families not recognised by Scudder (1979) but which contain species included by him within other families, are indicate by ? in this column. **^3^**Numbers in parentheses indicate the number of non-native species included in the total. Phytophagous species known only from indoor plants are not included. **^4^**Barcode Index Numbers, as defined by Ratnasingham and Hebert (2013). **^4A^**See figure 1 in Langor (2019) for a map of ecozones. **^5^**The baseline data for all groups is the most recent comprehensive checklist of Hemiptera in Canada (Maw et al. 2000). Sources given here are for subsequent additions to the known fauna. Collection abbreviation: CNCI, specimens in Canadian National Collection of Insects, Arachnids and Nematodes. **^6^**As the delineation of families of Psylloidea has changed since Scudder (1979), the number of species reported in 1979 for each family are not comparable to current totals with the exception of Triozidae. **^7^**In Scudder (1979), the count for Margarodidae included Matsucoccidae, Microphysoidea, and Xylococcidae; no Canadian species remain in Margarodidae. **^8^**Misspelled as Arthezidae in Scudder (1979). **^9^**In Scudder (1979), the count for Pseudococcidae included Putoidae. **^10^**In Scudder (1979), the count for Issidae included Acanaloniidae. **^11^**In Scudder (1979), the count for Cixiidae included Kinnaridae. **^12^**Treated under Fulgoridae in Scudder (1979); there are no Canadian species of Fulgoridae*s.str.***^13^**In Scudder (1979), the count for Cercopidae included Aphrophoridae and Clastopteridae. **^14^**Enicocephalidae was listed under Reduvioidea in Scudder (1979). **^15^**Ceratocombidae was treated as part of Dipsocoridae in Scudder (1979). **^16^**In Scudder (1979), the count for Anthocoridae included Lasiochilidae and Lyctocoridae. **^17^**Scudder (1979) reported on Miridae (600 spp.) and Isometopidae (1 spp.) separately; however, we have combined these counts for 1979 under Miridae. **^18^**Scudder (1979) reported on Reduviidae (13 spp.), Phymatidae (3 spp.), and Ploiariidae (10 spp.) separately; however, as these are all currently included in Reduviidae, we have combined the count for 1979. **^19^**Scudder (1979) reported on Aradidae (46 spp.) and Merizidae (1 spp.) separately; however, we have combined these counts for 1979 under Aradidae. **^20^**Scudder (1979) reported this family as Berytinidae. **^21^**In Scudder (1979), the count for Lygaeidae included Artheneidae, Blissidae, Cymidae, Geocoridae, Heterogastridae, Oxycarenidae, Pachygronthidae, and Rhyparochromidae. **^22^**Scudder (1979) reported on Pentatomidae (61 spp.) and Podopidae (2 spp.) separately; however, we have combined these counts for 1979 under Pentatomidae. **^23^**Scudder (1979) reported this family as Corimelaenidae.

A further 590 species are estimated to occur in the country, with the majority expected in the large families Aphididae, Cicadellidae, and Miridae. Estimates of the number of unrecorded species for the less diverse families is based mainly on known but undescribed species and presence of species in adjacent climatologically and ecologically similar parts of the United States, and known distributions of host plants. Molecular data and analysis of host plant usage provides evidence of additional cryptic diversity in the more speciose phytophagous groups. For some families, presence of unnamed clusters in the DNA barcode data suggests additional species, assuming that in most cases Barcode Index Numbers (BINs), as defined by Ratnasingham and Hebert (2013), correspond to one or more species (Gwiazdowski et al. 2015).

The classification used here follows Psyl’list (Ouvrard 2018) for Psylloidea, Aphid Species File (Favret 2018) for Aphidomorpha, ScaleNet (García Morales et al. 2016) for Coccoidea, Bartlett et al. (2018) for higher classification of Auchenorrhyncha, Bartlett et al. (2014) for species level delimitation in Fulgoromorpha, Dmitriev (2018) for species level delimitation in Cicadomorpha, and Henry (2017) for higher level classification of Heteroptera. There have been several changes in higher level classification since Scudder (1979). Homoptera, no longer recognised as a formal taxon, is now treated as two suborders, namely Sternorrhyncha and Auchenorrhyncha. Although not followed here, some authors (after Sorensen et al. 1995) separate the Auchenorrhyncha into suborders Clypeorrhyncha and Archaeorrhyncha. Among Sternorrhyncha, many new families of scale insects have been erected; species in Canada formerly included in Margarodidae are now dispersed among Matsucoccidae, Steingeliidae and Xylococcidae; Putoidae was formerly included in Pseudococcidae. Schemes for the subdivision of family Aphididae, such as that of Heie (1980) (and used in the Hemiptera checklist of Maw et al. 2000), have been proposed, but largely ignored in the absence of a clear consensus on the relationships among aphid subgroups. Within the Fulgoroidea, the Acanaloniidae, Dictyopharidae, and Kinnaridae are now recognised in Canada, with their species removed from the Issidae, Fulgoridae, and Cixiidae, respectively. The broadly constituted Cercopidae has been divided, with most Canadian species now placed in the Clastopteridae and Aphrophoridae. In the Heteroptera, most former lygaeid subfamilies have been given family status so that Lygaeidae of Scudder (1979) is now represented by eight families (Henry 1997); the further segregation of Ischnorhynchidae and Orsillidae (Sweet 2000) from Lygaeidae is not recognised here. Lyctocoridae and Lasiochilidae have been separated from Anthocoridae. On the other hand, Aradidae now includes Meziridae, Miridae includes Isometopidae, and Reduviidae includes Phymatidae and Ploiariidae.

The 419 non-native species of Hemiptera represents a significant proportion of the total fauna. In Aphididae, about 19% of the species are non-native. An overview of the non-native aphid fauna of North America was provided by Foottit et al. (2006) and updated by Skvarla et al. (2017). The non-native Heteroptera of Canada (about 7% of the total fauna) were treated by Scudder and Foottit (2006).

## Sternorrhyncha

Worldwide, the Sternorrhyncha are represented by about 18,690 species (Hardy 2018), with about 2950 species in North America (approximate composite number based on Foottit et al. 2006, García Morales et al. 2016, Skvarla et al. 2017, Mallory 2018, Ouvrard and Martin 2018). Currently, 1120 species of Sternorrhyncha are known from Canada compared to 834 in 1979, and it is expected that a further 215 species will be eventually found in the country (Table 1).

In Canada, the Aphididae and Adelgidae are relatively well known. Foottit and Maw (1997, 2014) contributed syntheses of the Yukon and grassland faunas of Aphididae. Aphids and adelgids are well represented by DNA barcodes (Foottit et al. 2008, 2009a, b), and, in general, barcode diversity in these groups corresponds well to morphological species concepts. However, several currently recognised aphid species are represented by more than one BIN, as defined by Ratnasingham and Hebert (2013). In two such cases, subsequent morphological analysis and addition of other genetic loci has resulted in the recognition of new species (Foottit et al. 2010, Foottit and Maw 2018). Conversely, members of several aphid species groups are not distinguishable by COI sequence divergence (Foottit et al. 2008).

The Psylloidea have not been subjected to extensive taxonomic or faunal analysis in Canada, except for work by Hodkinson (e.g., Hodkinson 1976) in British Columbia and Alberta. The identifiable forms of Coccoidea (adult female) and of Aleyrodidae (immatures) are sessile or subterranean and thus not captured by the usual general collecting methods. Consequently, the national fauna and regional distribution of species in these two groups are poorly known, and even limited efforts can yield new records. Kozár et al. (1989) identified several species of scale insects new to Canada based on brief collecting efforts in southern British Columbia. In a recent study of ant–sternorrhynch associations at a single grassland site in Alberta, two of the four species of Pseudococcidae found were newly recorded for Canada (Newton et al. 2011). The number of available BINs (see Table 1), largely based on untargeted sampling by the Biodiversity Institute of Ontario (University of Guelph), has indicated that current knowledge greatly under-represents the true fauna of Psyllidae and Aleyrodidae if BIN diversity can be considered a good approximation of species diversity in these groups.

## Auchenorrhyncha

Worldwide there are about 43,024 species of Auchenorrhyncha (Bartlett et al. 2018), but an estimate for North America is currently not available. In Canada, Hamilton (1997, 2014) analysed the cicadellid fauna of the Yukon and the Canadian Prairie Ecozone, respectively, Gareau (2008) documented that of Quebec, and Wilson (1997) treated the Yukon Delphacidae. The taxonomic status of several auchenorrhynch groups has been updated. A number of papers by Hamilton (e.g., Hamilton 1983, 1994, 1998) have revised many groups of Cicadellidae. As well, the large and difficult tribe Erythroneurini has been completely revised by Dmitriev and Dietrich (2007, 2009, 2010). The Cercopoidea were completely revised and a handbook published by Hamilton (1982). Progress on the Fulgoroidea includes a review of the North American fauna (Bartlett et al. 2014), which provides illustrated keys to all genera including the first comprehensive key to delphacid genera in the region. The known diversity of Canadian Auchenorrhyncha has increased since Scudder (1979), mostly as a result of taxonomic progress and improved knowledge of distributions. Currently, 1491 species are known from Canada compared to 1060 in 1979, and it is expected that a further 223 species will be eventually found in the country (Table 1). Most of the increases are in line with estimates of unrecorded species provided by Scudder (1979). The highest proportional increase is among the Cicadidae from nine to 21 recorded species; this increase is entirely due to improved knowledge of distribution (Hamilton 2010, Sanborn and Phillips 2013) as little taxonomic work has been done on the family in Canada. The highest numerical increase is among the Cicadellidae with an increase of 297 species; this is due to a combination of significant taxonomic research, greatly increased knowledge of distributions, and a small number of recently introduced species. We expect this family to hold the largest number of still unrecorded species (estimated at 150 species), particularly among the under-studied and under-collected subfamily Typhlocybinae.

Significant progress has been made in DNA barcoding of the Canadian Auchenorrhyncha. Data for 691 species have been released (Foottit et al. 2014, Gwiazdowski et al. 2015) and unpublished data for additional species is available in Barcode of Life Data System (Ratnasingham and Hebert 2007). A simple comparison between the number of BINs and recorded species suggests that more than half the Canadian species have been barcoded in most families, and an impressive 91% of the highly diverse Cicadellidae. However, caution is required in interpreting these numbers. Single BINs have been shown to include multiple morphologically distinct species in a number of cicadellid genera (Foottit et al. 2014). Conversely, preliminary examination of BINs for the Typhlocybinae suggests that single species may be represented by multiple BINs. Thus, the number of BINs may not be predictive of the number of distinct species within these groups.

## Heteroptera

There are about 45,254 described species of Heteroptera in the world. The most recent published comprehensive catalog for the group in North America (Henry and Froeschner 1985) includes 3834 species. About 1400 species are currently known to occur in Canada compared to 1171 in 1979, and it is expected that a further 151 species will be eventually added (Table 1). Most families of this suborder are relatively well known in the country. However, representatives of two families have been found in Canada only recently: Schizopteridae in 2010 (Scudder 2010a) and Aenictopecheidae in 2016 (Scudder and Štys 2016). Roch (2017) recently documented the Heteroptera of Quebec. Detailed analyses of the faunas of the Yukon, grasslands, Atlantic Maritime Ecozone and Montane Cordillera Ecozone have appeared (Scudder 1997, 2010b, 2011, 2014), and the aquatic and semiaquatic Heteroptera of Canadian peatlands and marshlands and the aquatic Heteroptera of the prairies and parklands were documented by Scudder (1987) and Scudder et al. (2010). Kelton (1980) provided a handbook of the Miridae of the Prairie Provinces. A major on-line database of the mirid fauna of North America (Schuh 2002–2013) includes records for a major portion of the holdings of the CNCI. DNA barcodes for the 334 species of Heteroptera drawn from CNCI (mainly Canadian species) were presented by Park et al. (2011) and reanalysed by Gwiazdowski et al. (2015).

The predicted number of Miridae constitutes the bulk of the estimate for Heteroptera overall, but this number is speculative and may be an underestimate if there are a significant number of undetected cryptic species within the more speciose genera.

## Summary and opportunities

Despite the significant increase in knowledge of Hemiptera in Canada since 1979, a substantial amount of the country’s biodiversity still awaits discovery. Some groups of Hemiptera are relatively well documented in Canada, while others are quite poorly known. However, even in the well-studied, but highly diverse phytophagous families (such as Aphididae and Miridae), there is probably unrecognised cryptic diversity associated with host plants and geographic variation. Several large genera in these families, such as *Lygus*, continue to present taxonomic difficulties (Schwartz and Foottit 1998) and opportunities for application of new approaches and technologies. Because many species of Hemiptera are current or potential pests, continuing research on detection, identification, quarantine and management of these groups will be required.
